# 2882. Impact of a Tele-Antimicrobial Stewardship Program at Two Small Community Hospitals in Partnership with an Academic Medical Center: 2 Years of Experience

**DOI:** 10.1093/ofid/ofad500.159

**Published:** 2023-11-27

**Authors:** Jennifer Ross, Aditya Chandorkar

**Affiliations:** M Health Fairview - University of Minnesota Medical Center, Minneapolis, MN; University of Minnesota, Minneapolis, Minnesota

## Abstract

**Background:**

Community and rural hospitals often lack resources for infectious diseases (ID) specialists. Tele-antimicrobial stewardship programs (TASPs) have emerged as a method for health systems to meet regulatory requirements. We analyzed the impact of a fully remote TASP at two small community hospitals in partnership with an academic medical center over a two-year period.

**Methods:**

A TASP, co-led by an ID physician and ID pharmacist, was implemented at a 21-bed hospital in Princeton, MN, and 49-bed hospital in Wyoming, MN, in August 2020. Figure 1 outlines daily TASP workflows in which the ID physician and ID pharmacist were located at an academic medical center. Frequently encountered restricted antimicrobial agents included vancomycin (intravenous and enteral), piperacillin-tazobactam, cefepime, meropenem, ertapenem, micafungin, and remdesivir. Antimicrobial stewardship interventions were tracked monthly. Restricted antimicrobial days of therapy per 1000 patient days (DOT/1000 PD) mean averages two years pre- and post-implementation were compared. Annual antimicrobial expenditures were followed.

**Figure:**
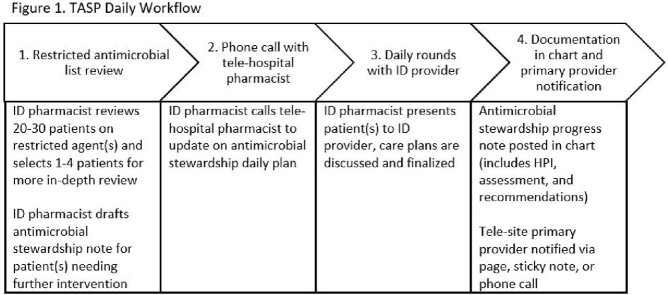

**Results:**

For the first two TASP years (8/1/2020 to 7/31/2022), a total of 789 antimicrobial interventions were made with 85.6% being accepted. Each site boasted similar TASP intervention acceptance rates, 85% for the Princeton hospital and 86% for Wyoming (Figure 2 and Table 1). Restricted antimicrobial use trended down from 141.97 to 113.97 DOT/1000 PD at Princeton. A smaller decrease from 106.3 to 103.12 DOT/1000 PD was seen at Wyoming. Annual antimicrobial costs per total patient days also decreased (Figure 3). Princeton hospital’s annual antimicrobial expenditures per total patient days fell from $18.89 in 2019 (pre-implementation) to $6.64. Wyoming showed a reduction in antimicrobial costs per total patient days by decreasing from $11.20 to $5.36.

**Figure:**
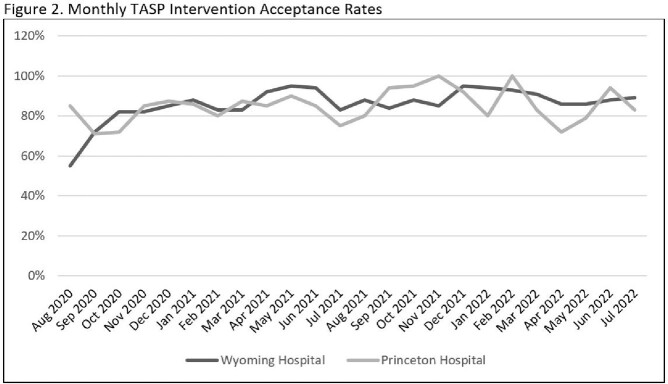

**Figure:**
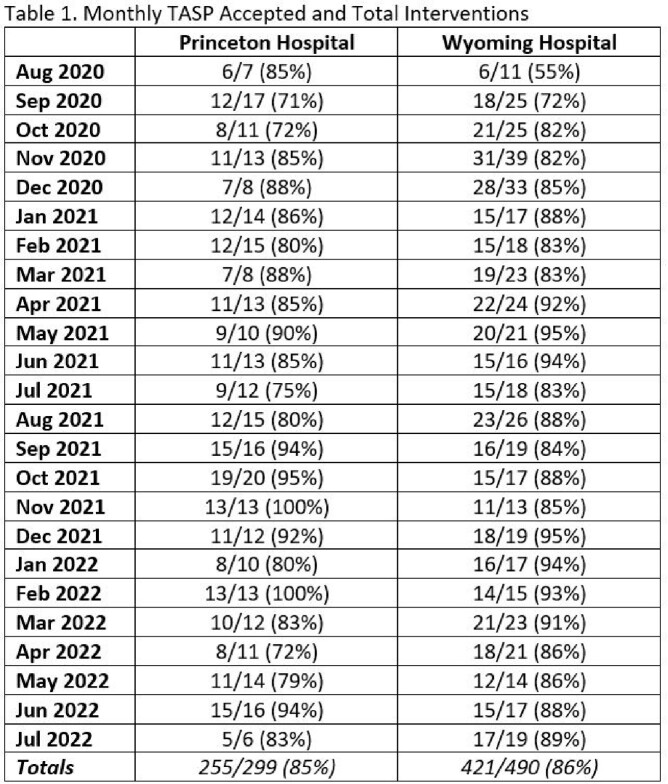

**Figure:**
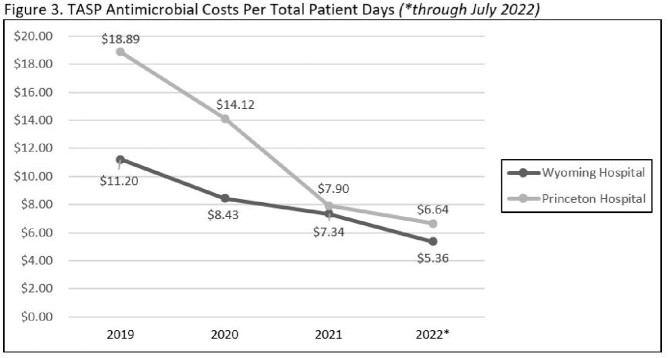

**Conclusion:**

A fully remote TASP in partnership with an academic medical center for two small community hospitals resulted in a rapid increase in the rates of accepted interventions. These high rates were sustained over two years. Restricted antimicrobial use and antimicrobial costs trended down. Consistent communication with tele-hospital physicians and pharmacists helped establish trusting relationships.

**Disclosures:**

**All Authors**: No reported disclosures

